# Real-life isoniazid and rifampicin plasma concentrations in children: a tool for therapeutic drug monitoring of tuberculosis

**DOI:** 10.1186/s12879-021-06764-7

**Published:** 2021-10-21

**Authors:** Chiara Tersigni, Giulia Boiardi, Lorenzo Tofani, Elisabetta Venturini, Carlotta Montagnani, Barbara Bortone, Leila Bianchi, Elena Chiappini, Maria Iris Cassetta, Stefania Fallani, Andrea Novelli, Luisa Galli

**Affiliations:** 1 Post Graduate School of Pediatrics, University of Florence, Florence, Italy; 2 University of Florence, Florence, Italy; 3 Department of Health Sciences, Clinical Pharmacology and Oncology Section, University of Florence, Florence, Italy; 4 Infectious Diseases Unit, Meyer Children’s University Hospital, Florence, Italy; 5 Department of Health Sciences, University of Florence, Anna Meyer Children’s University Hospital, Florence, Italy

**Keywords:** Therapeutic drug monitoring, Children, Tuberculosis, Treatment compliance, Isoniazid, Rifampicin

## Abstract

**Background:**

Low plasma levels of first-line antitubercular drugs can be counted among the main causes of poor response to antitubercular therapy, and therapeutic drug monitoring has been proposed as a method to promote tailored treatments for both child and adult patients. The main aim of the study was to evaluate serum concentrations of isoniazid (INH) and rifampicin (RIF) and to investigate reasons for sub-therapeutic plasma concentrations in order to fix dosages.

**Methods:**

Children with TB were prospectively enrolled from January to August 2019. Two venous blood samples were collected (the first at least 15 days after the beginning of antitubercular treatment, and the second between 1 and 8 weeks later). Plasma concentrations were determined by a validated high-performance liquid chromatography method.

**Results:**

In all, 45 children were included. Seventy blood samples for INH plasma concentration were collected between 120 and 240 min after drug intake. Adjusting for dose (mg/kg/day) and time of INH administration, when considering three different age groups (≤ 2 years, 2–12 years, > 12 years), a statistically significant lower INH plasma concentration was observed in younger children compared to the older age groups in the multivariate analysis (p < 0.001 and p < 0.001). A total of 68 blood samples were evaluated for RIF concentrations. Both for INH and RIF a statistically significant lower plasma concentration was also observed in adolescents (p < 0.001). Fifteen children (15/45, 33%) presented drug concentrations under the referral therapeutic range.

**Conclusions:**

Based on our findings, monitoring patients’ drug plasma concentrations in children under 2 years of age and in adolescents can make treatment more patient-tailored.

## Background

Tuberculosis (TB) is one of the leading causes of human morbidity and mortality from a curable infectious disease and remains a major global health problem [1]. Despite the fact that the incidence rate has been decreasing since 2000, TB still ranks among the top ten causes of death [1, 2]. Furthermore, childhood TB can be considered a sentinel of the disease spreading in the community, mostly because children are at a significantly higher risk than adults of developing the active form of the disease [2, 3]. As a result, pediatric TB deserves prompt identification and treatment [4].

Several studies have investigated the different causes of antitubercular treatment failures. The lack of adherence to therapeutic regimens, drug resistant strains, the presence of comorbidities and the variability in the pharmacokinetics of first-line TB drugs were identified as the most important factors [5, 6]. Furthermore, it is estimated that nearly 15% of patients treated for active and latent TB present a poor response to treatment regimens [5]. A large inter-patient variability of plasma levels of isoniazid (INH), with plasma concentrations frequently below the expected therapeutic range has been observed in most studies [5–7]. Low plasma levels of first-line antitubercular drugs can be counted as being among the main causes of poor response to therapy. Furthermore, a delayed or insufficient treatment response can lead to prolonged therapy, and consequently to the development of resistant strains or possible relapses of the disease [8, 9].

However, early biomarkers which can identify patients requiring prolonged treatments or predict treatment efficacy have yet to be found [8]. For this reason, therapeutic drug monitoring (TDM) has been proposed as a method for evaluating patient compliance with therapy, in order to fix drug doses on the basis of plasma concentrations. These strategies can make therapies more patient-tailored and can avoid the development of resistant strains, which might result from drug concentrations far below therapeutic values, or they can prevent the occurrence of adverse reactions related to supra-therapeutic levels [10, 11].

In the literature, there is a lack of published studies regarding plasma concentration of INH and rifampicin (RIF) in children, especially under 2 years of age, with referral therapeutic ranges often based on adult studies [12, 13]. However, these studies are fundamental to guiding recommended pediatric TB drug dosages. In fact, in 2010 the WHO revised its guidelines to suggest higher doses of first line antitubercular drugs (INH 10–15 mg/kg/day versus 4–6 mg/kg/day and RIF 10–20 mg/kg/day versus 8–12 mg/kg/day), based on studies conducted on older children or adults [14–17].

The present study aims to evaluate serum concentrations of INH and RIF in children treated for latent and active TB. It will investigate reasons for sub-therapeutic plasma concentrations of INH and RIF in order to fix dosages when necessary, promote a patient-tailored treatment and prevent treatment failure and the onset of drug resistant strains.

## Methods

Children (0–18 years) with active and latent TB treated with INH and RIF were prospectively enrolled at the Pediatric Infectious Diseases Unit of a tertiary care pediatric university hospital located in Florence, Italy (Anna Meyer Children’s University Hospital) from January 2019 to August 2019. An average of 45 latent and 25 active cases a year are evaluated in the TB clinic. The following data were collected in the present prospective observational study: demographic characteristics and treatment information (drug type, dosage, therapy start and end dates, time of drug intake, adverse events and associated drugs). All data were recorded in the study database following the international standards for the protection of privacy and personal information. The study was approved by the ethical committee (20/12/2018-188/2018).

### Study design

Children were included in the present study within their first clinical examination, at least 15 days after the beginning of antitubercular therapy (achieved steady state of INH and RIF) [15]. During the first evaluation, a clinical assessment was made of the reason for investigations and the presence or absence of TB drug-related side effects. Microbiological data (in cases of active TB) were collected. Routine blood exams were carried out to detect any adverse reactions (full cell blood count, creatinine, c-reactive protein and serum transaminases). A venous blood sample (4 ml in EDTA) was added to the routine blood tests at least 1 week after the beginning of antitubercular treatment (median 54 days, IQR: 30.7–94.7).

The same sample was taken in one of the following scheduled evaluations, between 1 and 8 weeks after the first. Blood samples were centrifuged immediately after collection and the plasma was separated and stored at − 20 °C until analysis. Concentrations were determined by a validated High Performance Liquid Chromatography (HLPC) method [18, 19].

INH samples and calibration standards were extracted with Amicon Ultra 0.5 ml 10 K filters and 180 µl of the filtrate was injected into the column. A symmetry C18 column (250 × 4.6 mm, 5 µm; Waters, Milford, MA, USA) was used for the INH analysis. INH was isocratically eluted at 263 nm at a constant flow rate of 1.0 ml/min. The mobile phase was made up of ammonium acetate 50 mM and methanol, 90:10 (v/v). The column was kept at room temperature for the entire duration of the analysis. Calibration curves were performed in a pool of plasma with the addition of INH at the following concentrations 8, 4, 2, 1, 0.5, 0.25 mg/l [18].

RIF samples and calibration standards were extracted with acetonitrile (33:67 v/v) in polypropylene tubes (Sarstedt, Leicester, UK). The mixture was briefly vortex-mixed, kept for 10 min at room temperature and then centrifuged at 1200×*g* for 10 min. The supernatant was dried, the dry residue reconstituted with 0.5 ml of mobile phase, and 180 µl was injected into the column. A Luna C18 column (250 × 4.6 mm ID, 5 µm; Phenomenex, Torrance, CA, USA) protected by an adequate pre-column was used for RIF analysis. RIF was isocratically eluted at 254 nm at a constant flow rate of 1.2 ml/min. The mobile phase consisted of potassium dihydrogen phosphate 0.05 M (pH 2.6) and acetonitrile 55:45 (v/v). The column was kept at room temperature for the entire duration of the analysis. Calibration curves were carried out in a plasma pool with the addition of RIF at the following concentrations 16, 8, 4, 2, 1, 0.5, 0.25 mg/l [19].

Both INH and RIF assays were linear (*r* = 0.9997) in the 0.1–25 mg/l concentration range. Precision and accuracy coefficients of variation were between 2.4 and 7.3%; intra-day accuracy was in the range of 95–106.5%.

On the basis of INH and RIF pharmacokinetic parameters, samples were collected between a minimum of 2 and a maximum of 8 h after drug administration. Blood samples evaluated for the statistical analysis were obtained between 120 and 240 min after drug intake [20]. Patients with unknown sampling times and those receiving the study drugs more than 8 h before sampling were excluded from the analysis.

### Definitions

Active TB cases were diagnosed according to two categories:

1. Definite TB: children with *Mycobacterium tuberculosis* cultured or detected by microscopy or molecular methods from gastric aspirate culture or sputum;
2. Probable TB: absence of microbiological confirmation but presence of the following criteria: abnormal radiography and/or computed tomography scan consistent with lung TB, positive clinical response to TB therapy, clinical signs and symptoms of active TB, and either a history of TB contact or travel to a TB-endemic country within the last 24 months [21].

Latent TB was diagnosed in children with a positive tuberculin skin test and/or interferon-γ release test, without clinical or radiological signs of active disease [19].

According to available studies conducted in children, the INH considered reference range was 3–5 μg/ml after 2 h and 1–3 μg/ml after 4 h. The RIF considered therapeutic range was 8–24 μg/ml after 2 h based on two studies conducted in adults. [22–25].

### Statistical analysis

Statistical analysis was performed using SAS 9.3. In order to evaluate the normal distribution of continuous variables, the Shapiro–Wilk test was used. For each continuous variable, the mean and standard deviation (SD) or median and interquartile range (IQR) (according to the Shapiro–Wilk test result) is reported. For categorical variables, absolute frequencies and percentages are reported for each category. To evaluate the association between a single RIF or INH dosage and each covariate, a simple linear regression was used. In order to assess associations between all RIF or INH dosages and patient characteristics (age, sex and ethnicity) a simple and multiple GEE (generalized estimating equation model) linear model was used, including all characteristics (such as age, sex and ethnicity). The significant level was set at 5%.

## Results

Children diagnosed with active or latent TB were enrolled in the present study. Overall, 45 children were included (19 with active TB and 26 with latent TB) with a median age of 82 months [IQR 38–148]. The anagraphic data and the main characteristics of enrolled children are reported in Table 1.

**Table 1:** General characteristics of enrolled children

Patients characteristics	Latent TB n = 26	Active TB n = 19	All n = 45
Sex			
Male	17/26 (65.4%)	15/19 (78.9%)	32/45 (71.1%)
Female	9/26 (34.6%)	4/19 (21.1%)	13/45 (28.9%)
Age (months)—median [IQRs]	101 [64–187]	43 [22–134]	82 [38–148]
Reason for investigation			
Adoption/immigrant screening	11/26 (42.3%)	0/19	11/45 (24.4%)
Household contact	12/26 (46.2%)	6/19 (31.6%)	18/45 (40%)
Symptomatic	0/26	11/19 (57.9%)	11/45 (24.4%)
Not household contact	3/26 (11.5%)	2/19 (10.5%)	5/45 (11.2%)
Ethnicity			
Caucasian	15/26 (57.7%)	10/19 (52.6%)	25/45 (55.5%)
African	0/26	4/19 (21%)	4/45 (8.9%)
Asian	3/26 (11.5%)	2/19 (10.5%)	5/45 (11.1%)
South American	8/26 (30.8%)	3/19 (15.8%)	11/45 (24.5%)
Type of TB			
Microbiological confirmed	–	9/19 (47.3%)	–
No microbiological confirmed	–	11/19 (57.9%)	–

*TB* tuberculosis; *IQR* interquartile range

### Treatment regimen and side effects in children with active and latent TB

Children with active TB were 19/45 (42.2%), of which 17/19 (89.5%) were treated with a four-drug regimen comprised of INH, RIF, pyrazinamide (PZN) and ethambutol (ETB), and 2/19 (10.5%) with RIF, PZN and ETB (due to the onset of INH resistance). Eleven out of 19 children (57.9%) were evaluated because the presence of TB symptoms (prolonged fever, persistent cough), and 9/19 because of contact with active TB cases. Four children (21%) presented vomiting and abdominal pain as side effects of treatment, with transaminase values within the physiological range in the totality of patients. No side effects caused treatment discontinuation. The majority of patients had pulmonary TB (63.1%) with *Mycobacterium tuberculosis* cultured or detected by microscopy or molecular methods from gastric aspirate in about half of the cases (9/19, 47.3%).

Patients diagnosed with latent TB were 26/45 (57.8%). Twenty-one (80.8%) were treated with a two-drug regimen comprised of INH and RIF, and 4/26 (15.4%) with INH alone. The remaining patient was treated with RIF due to the onset of INH resistance.

The majority of these patients presented no adverse events to the antitubercular therapy. Only two patients (7.7%) reported vomiting. The routine blood exams showed transaminase values within the physiological range in 24/26 (92.3%) patients and slightly elevated in one patient. Only one case of hypertransaminasemia was reported, which led to the treatment being suspended for a month (plasma concentrations of INH and RIF were both in the referral therapeutic range).

### Isoniazid plasma concentrations

Overall, 43 blood samples were collected during the first evaluation and 37 during the second scheduled clinical assessment. The median regimen duration of children treated with INH was 54 (IQR 33–95) days and 85 (IQR 19–64) days during the first and the second evaluations respectively.

**Fig. 1:**
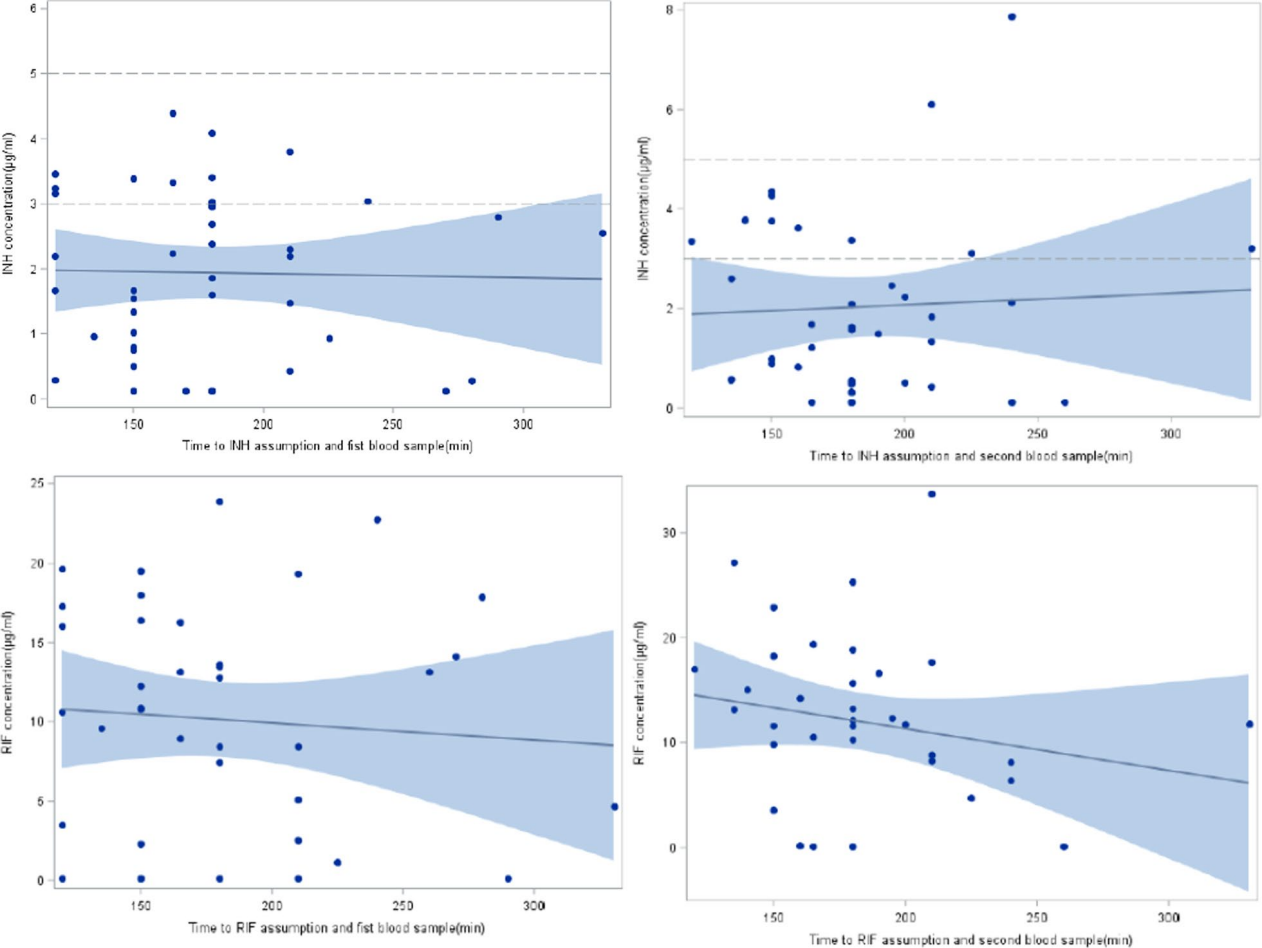
Isoniazid and rifampicin times of samples collection

Overall, 73 blood samples were collected between 120 and 240 min after drug intake. Time of INH samples collection is reported in Fig. 1.

**Fig. 2:**
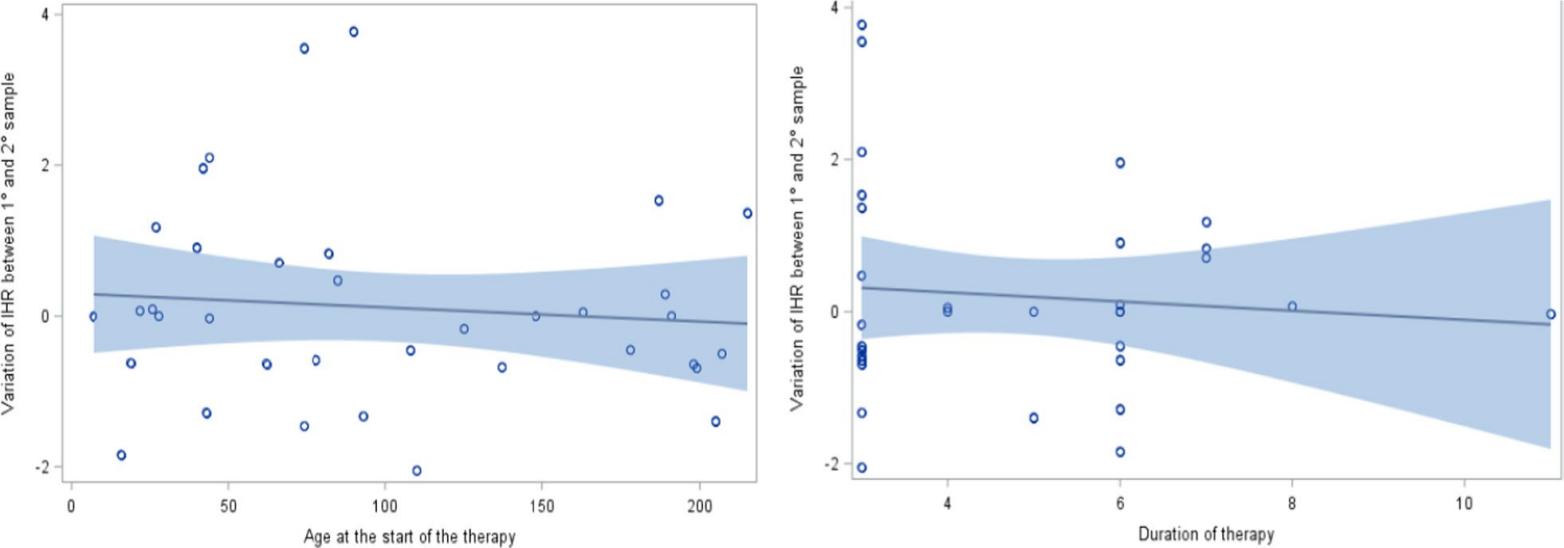
Variation of isoniazid level and gender between the first and the second blood sample

**Fig. 3:**
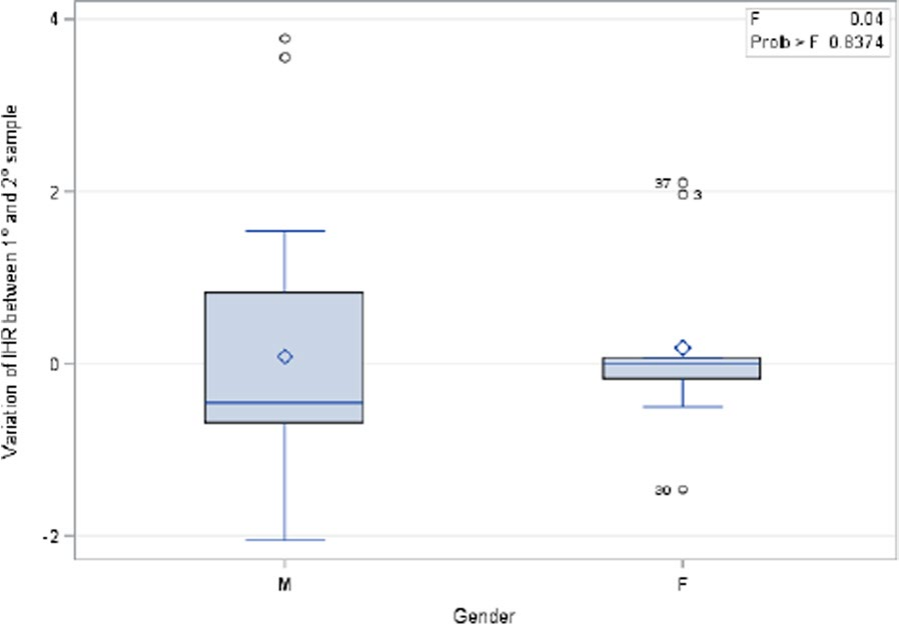
Analysis of simple regression (blue line) and its 95%CI (blue band) for variation of isoniazid level and age at the beginning of therapy (figure on the left) and duration of therapy (figure in the right) between the first and the second blood sample

No statistically significant variations of INH concentration in the first and second blood samples were detected, when age at the beginning of INH treatment, gender and duration of therapy (p = 0.57, p = 0.62 and p = 0.83) (Fig. 2, 3) were taken into account.

Mean INH plasma concentrations 2–4 h after drug intake were 1.95 μg/ml (SD ± 1.25) in the first blood sample and 2.04 μg/ml (SD ± 1.79) in the second, with an overall mean of 1.99 μg/ml (SD ± 1.51).

Since duration of INH administration showed no statistically significant correlation with INH plasma concentration both in the first and in the second evaluations (p = 0.77 and p = 27, respectively), patient blood samples were analyzed overall.

With regard to reasons for investigation (symptomatic vs contact of TB index case) and type of TB (microbiological-confirmed vs. non-microbiological-confirmed), no statistically significant differences of INH concentrations were observed in the two groups (p = 0.95 and p = 0.89, respectively).

When adjusting for the dose (mg/kg/day) of administered INH, drug concentrations showed a statistically significant difference by sex, being higher in male than female (median 2.22 μg/ml IQR 1.13–3.29 vs 0.75 μg/ml IQR 0.44–1.67; p = 0.0115) patients. However, when considering different age groups (≤ 12 years vs > 12 years), only INH concentrations in older males statistically differed from those found in females (p = 0.04).

In respect of patients’ place of birth, no statistically significant differences were observed (p = 0.09).

Adjusting for the dose (mg/kg/day) and for the time of INH administration, when considering three different age groups (≤ 2 years, 2–12 years, > 12 years), a statistically significant lower INH plasma concentration was observed in younger children compared to the older age groups (p = 0.001 and p = 0.0024). This finding was also confirmed in the multivariate analysis (p < 0.001 and p < 0.001) (Table 2).

**Table 2:** Univariate and multivariate analysis of isoniazid concentration considering age, gender and ethnicity

	n (%)	Median plasma concentration μg/ml (IQR)	Univariate analysis p	Multivariate analysis p
Age groups (years)				
≤ 2 years	10 (13.7)	0.78 (0.43–1.86)		
2–12 years	42 (57.5)	2.27 (1.34–3.39)	**0.0024**	** < 0.001**
> 12 years	21 (28.8)	1.55 (0.5–2.47)	** < 0.001**	** < 0.001**
Gender				
Male	52 (71.2)	2.22 (1.13–3.29)	**0.01**	**0.056**
Female	21 (28.8)	0.75 (0.44–1.67)		
Ethnicity				
African	5 (6.8)	2.61(2.20–3.24)	0.90	0.238
Asiatic	7 (9.6)	0.94 (0.44–4.35)	0.52	0.88
South American	14 (19.2)	2.05 (0.52–2.96)	0.74	0.86
Caucasian	47 (64.4)	1.67 (0.80–3.12)		

Value reported in bold those statistically significant (*p* < 0.05)

*IQR* interquartile range

A statistically significant lower INH plasma concentration was also observed in children aged > 12 years (p < 0.001).

### Rifampicin plasma concentrations

Thirty-nine blood samples were collected during the first evaluation and 37 during the second scheduled clinical assessment. Overall, 68 blood samples were evaluated for RIF plasma concentration. No statistically significant differences by sex or patient place of birth were observed (p = 0.24 and p = 0.92, respectively).

Mean RIF plasma concentrations 2–4 h after drug intake were 10.13 μg/ml (SD ± 7.22) in the first blood sample and 11.67 μg/ml (SD ± 8.23) in the second, with an overall mean of 10.88 μg/ml (SD ± 7.72).

**Fig. 4:**
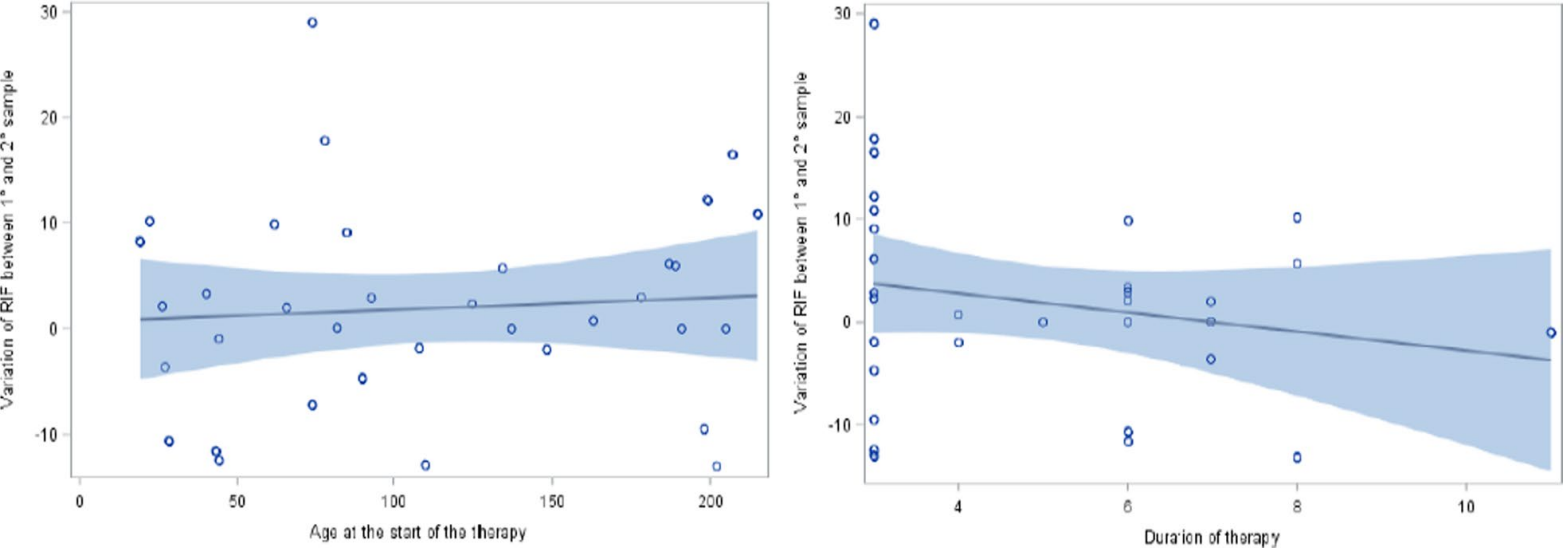
Variation of rifampicin level and gender between the first and the second blood sample

**Fig. 5:**
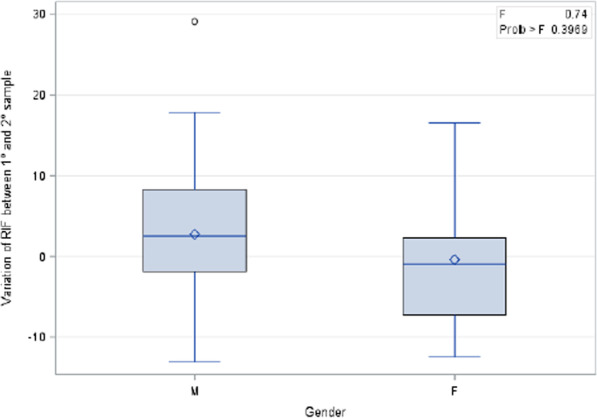
Analysis of simple regression (blue line) and its 95%CI (blue band) for variation of rifampicin level and age at the beginning of therapy (figure on the left) and duration of therapy (figure on the right) between the first and the second blood sample

In addition, no statistically significant variations of RIF concentration in the first and in the second blood sample were detected when considering age at the beginning of RIF treatment, sex and duration of therapy (p = 0.64, p = 0.24 and p = 0.39, respectively) (Figs. 4 and 5).

Time of RIF samples collection is reported in Fig. 1.

Because duration of RIF administration showed no statistically significant correlation with RIF plasma concentration, both in the first and in the second evaluation (p = 0.94 and p = 0.26, respectively), blood samples of patients were analyzed overall.

Concerning reasons for investigation (symptomatic vs contact of TB index case) and type of TB (microbiological-confirmed vs. non-microbiological-confirmed), no statistically significant differences of RIF concentrations were observed in the two groups (p = 0.3 and p = 0.051, respectively).

Adjusting for the dose (mg/kg/day) of administered RIF, no statistically significant differences were observed between the sexes, both in the univariate and multivariate analyses (p = 0.24) (Table 3).

**Table 3:** Univariate and multivariate analysis of rifampicin concentration considering age, gender and ethnicity

	n (%)	Median plasma concentration μg/ml (IQR)	Univariate analysis p	Multivariate analysis p
Age groups (years)				
≤ 2 years	6	10.46 (0.13–13.16)		
2–12 years	41	13.17 (8.45–17.30)	0.11	** < 0.001**
> 12 years	21	8.96 (0.13–12.35)	0.61	0.15
Gender				
Male	51	10.63 (3.51–16.28)	0.24	0.31
Female	17	12.16 (10.29–17.03)		
Ethnicity				
African	5	17.30 (13.16–18.90)	**0.02**	** < 0.01**
Asiatic	7	14.26 (7.44–18.32)	**0.03**	0.05
South American	13	12.16 (10.87–16.67)	**0.04**	0.05
Caucasian	43	9.6 (0.19–15.72)		

Value reported in bold those statistically significant (p < 0.05)

*IQR* interquartile range

Regarding patients’ place of birth, a statistically significant higher plasma concentration was observed in Africans compared to other ethnicities in the multivariate analysis (p < 0.001).

Adjusting for the dose (mg/kg/day) and for the time at RIF administration, when considering three different age groups (≤ 2 years, 2–12 years, > 12 years), a statistically significant higher RIF plasma concentration was observed in children aged 2–12 years (p < 0.001).

### Children with subtherapeutic concentrations of INH and RIF

Fifteen children (15/45, 33%) presented drug concentrations under the referral therapeutic range either for INH or RIF.

With particular regard to INH, seven children had subtherapeutic values of INH both in the first and in the second blood samples. For RIF, 4 children had subtherapeutic values of the drug, both in the first and in the second blood samples.

Three children had permanent (both in the first and in the second blood samples, and for both drugs) subtherapeutic concentrations of INH and RIF.

One child was an 11-year-old Italian boy, treated for active TB with a four-drug regimen, with a median dose of INH of 8.82 mg/kg/day and of RIF 17.64 mg/kg/day.

The other two children were two brothers from Romania treated for latent TB. Over the course of their scheduled evaluations, reduced compliance by the family was observed.

## Discussion

To our knowledge, this is one of the few pediatric studies which aims to apply a TDM approach to antitubercular treatment through the evaluation of INH and RIF plasma concentrations using an HPLC method. Although TDM is recognized to be an important tool for TB treatment, it has not yet been applied in the clinical routine, and is reserved for patients with a more serious illness or non-responders [24].

Overall, 45 children diagnosed with latent or active TB were prospectively enrolled in the present study. Mean plasma concentration 2–4 h after drug intake was 1.99 μg/ml (SD ± 1.51) for INH and 10.88 μg/ml (SD ± 7.72) for RIF. Fifteen children (33%) presented drug concentrations under the referral therapeutic range either for INH or RIF. Both for INH and RIF, no correlation with duration of therapy was observed (p = 0.89 and p = 0.39 respectively).

Differently from our results, in a study conducted by Fahimi et al., a statistically significant correlation between INH therapy duration and plasma concentrations was observed (p < 0,001). In fact, INH plasma concentration progressively increased with therapy duration. [24].

In the present study, no statistically significant differences were observed with regard to reason for investigation, patient and parent place of birth, or associated drugs and diagnosis. Regarding the correlation between low plasma concentration of INH and sex, a statistically significant difference was observed in our study. In fact, INH plasma concentration was found to be higher in older males (≥ 12 years) than females (p = 0.004).

In this regard, in 2003, Ray et al. analyzed INH plasma concentrations in 85 adults treated with INH and observed significantly higher values in the INH plasma concentrations in males compared to females. They attributed the difference to the fact that females presented a higher dose when normalized for bodyweight [22]. This result can be considered in line with our finding, based on the fact that INH plasma concentration was found to be significantly higher in adolescent males compared to females. However, accurate comparisons between these results in adults and current findings in children are difficult, in light of the impact of dosages and the pharmacokinetic differences between these two categories. In contrast, Prahl et al. in 2014, investigated the clinical correlation with 2-h plasma concentrations of INH, RIF, PZN and ETB in 35 adults. No statistically significant difference was observed regarding drug formulation, dosage, therapy duration and gender but a correlation between low plasma levels and therapy failure was observed. In fact, five out 35 patients experienced disease relapse or died during treatment. Compared with enrolled patients with a successful treatment outcome, cases which experienced therapy failure presented lower INH plasma concentrations [26].

This finding was confirmed by a study involving 39 children with active TB aged 2–16 years conducted by Ranjalkar et al. in 2017. No significant effect of sex, age, body weight, type of TB, nutritional status and treatment regimen on plasma concentrations of either INH or RIF was observed [27].

Furthermore, Magis-Escurra et al., in a study on 41 adults, observed no statistically significant difference when considering sex, age and body mass index (BMI) on INH and RIF plasma concentrations [28].

A statistically significant difference was also observed between different age groups (≤ 2 years, 2–12 years, > 12 years) regarding INH plasma concentration. In fact, INH levels were statistically significantly lower in younger children compared to the older age groups in both the univariate and multivariate analysis (p < 0.001 and p < 0.001).

This finding was also highlighted in a study conducted by Schaaf et al. in 64 children. Authors reported a significant age-related decline in the elimination rate constant. In fact, younger children eliminated INH faster than older children, and children as a group faster than adults [17].

In 2009 McIlleron et al. evaluated plasma concentrations of antitubercular drugs in 56 southern African children. Peak plasma concentrations were below the recommended therapeutic ranges in almost 70% of patients. They reported that younger children required higher doses of INH per kilogram of body weight in order to reach INH plasma concentrations similar to those in adults [29]. These results can be attributed to the faster hepatic metabolism of younger children with an increased drug clearance normalized for body weight [30].

In addition to pharmacokinetic differences, lower drug plasma concentrations in younger children may be also related to the absence of pediatric formulations of antitubercular drugs, especially for INH. In this regard, Pouplinet al. investigated INH, PZN and RIF content uniformity in split tables used in the treatment of childhood TB. The authors reported that the content uniformity of antitubercular drugs in solid formulations is altered when the whole tablet is broken. [31].

A statistically significant lower plasma concentration of INH and RIF was also observed in older children (> 12 years) compared to the age group of children aged 2–12 years (p < 0.001). This finding could be related to the lower oral medication adherence of adolescents, described in the literature both for TB treatment regimens and for prolonged treatments for chronic conditions [32–34]. Several strategies have been proposed based on medication adherence apps, wirelessly observed therapy, video directly observed therapy, short message service reminders and adolescent education and counselling services, with a positive impact on treatment adherence [35–39].

When patient place of birth is taken into account, a statistically significant higher plasma concentration was observed in Africans compared to other ethnicities in the multivariate analysis (p < 0.001). The influence of race and genetic polymorphisms on drug concentration has been evaluated in the literature to support tailored treatments based on genetic diversity [40–42].

This study presents several limitations. Due to the real-life design of the study, conducted during scheduled clinical evaluations, we were not able to calculate area under the curve (AUC) and maximum or minimum plasma concentrations. No information regarding fasting conditions before drug intake was available. Moreover, no information regarding cure rates and TB treatment outcomes is available. A clinical follow-up is fundamental to determining whether low INH and RIF plasmatic levels are associated with treatment failures and relapses. Larger studies, especially in children under 2 years of age are needed to confirm these findings.

## Conclusions

About 30% of children with plasma concentrations of INH or RIF under therapeutic levels were identified in the present study, with lower INH plasma concentrations in younger children. Low drug plasma concentrations can lead to poor therapy outcomes and to the onset of drug-resistant strains. Monitoring patients’ drug plasma concentrations in order to fix dosages where necessary, especially in children under 2 years of age, can make treatment more patient-tailored. Further studies are necessary in order to apply TDM in children, to identify precise therapeutic reference ranges, based on different age groups and on genetic characteristics.

## Data Availability

The datasets used and/or analyzed during the current study are available from the corresponding author on reasonable request.

## Abbreviations

TB: Tuberculosis
WHO: World Health Organization
INH: Isoniazid
RIF: Rifampicin
TDM: Therapeutic drug monitoring
HIV: Human immunodeficiency virus
HLPC: High Performance Liquid Chromatography
SD: Standard deviation
IQR: Interquartile range
GEE: Generalized estimating equation model
PZN: Pyrazinamide
ETB: Ethambutol
